# Neotropical ants are at greater risk from global warming in savanna than in adjacent forest

**DOI:** 10.1002/ecy.70413

**Published:** 2026-05-13

**Authors:** Lino A. Zuanon, Karen C. Neves, Alan N. Andersen, Heraldo L. Vasconcelos

**Affiliations:** ^1^ Instituto de Biologia Universidade Federal de Uberlândia Uberlândia Brazil; ^2^ Research Institute for the Environment and Livelihoods Charles Darwin University Darwin Northern Territory Australia

**Keywords:** Cerrado, climate change, Formicidae, microclimatic variation, physiological traits, vertical stratification

## Abstract

Determining how the thermal tolerances of species are related to climatic conditions at multiple spatial scales can improve our understanding of species distributions and their vulnerability to climate change. We compare the warming tolerances—a metric of warming vulnerability—of arboreal and ground‐dwelling ants from savanna and adjacent semideciduous forest in Brazil's Cerrado. Warming tolerance was estimated using the difference between an ant's upper thermal limit and a thermal index of its habitat and stratum. We also evaluated if differences in the upper and lower thermal limits of Cerrado ant assemblages conform to the thermal adaptation (TAH) and the niche asymmetry (NAH) hypotheses. We found that the mean critical thermal maximum (CT_max_) and range (CT_range_) were higher for ants in savanna than in forest, a pattern that is consistent with the TAH as savanna had higher maximum and more variable air temperatures. However, arboreal ants had lower CT_min_ than those on the ground despite the similarities in minimum temperatures between the two strata. CT_max_ was lower for ground than for arboreal ants even though in the savanna (but not in the forest) average maximum air temperatures on the ground were 2°C higher than in trees. Further, the greater heat tolerance of savanna ants was less than the ~7°C difference at the ground stratum in mean maximum temperatures between savanna and forest. A moderate phylogenetic signal was found for CT_max_, CT_min_, and CT_range_. However, accounting for phylogeny did not change our results. Our key finding is that vulnerability to global warming cannot be adequately predicted based on heat tolerance alone—species having a similar CT_max_ can have very different vulnerability to global warming because of differences in exposure to direct insolation of their preferred habitat or stratum. In our study system, savanna ground ants are more vulnerable to global warming compared to ants living on the forest floor or to arboreal ants more generally. This may have important implications for conservation of the Brazilian savanna ant fauna since most Cerrado species, including several endemics, nest and forage on the ground.

## INTRODUCTION

In recent years, a growing body of research has focused on understanding the extent to which different plant and animal assemblages may become vulnerable to global warming (e.g., Anderson et al., 2022; Diamond, Nichols, et al., 2012; Diamond, Sorger, et al., 2012; Parr & Bishop, 2022; Walters et al., 2018). Warming vulnerability is often estimated using metrics that evaluate the risk of an organism in encountering environmental temperatures that are higher than its maximum critical thermal limit. One such metric is warming tolerance, defined as the difference between the maximum critical thermal limit of a species and a thermal index of its preferred habitat (Deutsch et al., 2008; Diamond, Sorger, et al., 2012; Huey et al., 2009; Leahy et al., 2022). A predictive understanding of warming tolerances thus requires an understanding of the extent to which the critical thermal limits of different taxa correlate with climatic gradients at different spatial and temporal scales (Diamond & Chick, 2018; Diamond, Sorger, et al., 2012; Huey et al., 2009; Nascimento et al., 2022; Sunday et al., 2012).

Differences in physiological thermal limits between species living in different regions or habitats often reflect the differences in the climatic conditions they experience (Roeder et al., 2021). According to the thermal adaptation hypothesis (TAH), the thermal limits of a species should track the temperatures it is exposed to, given that maintaining thermal limits that are greater than required would be unnecessarily costly (Kaspari et al., 2015). In other words, the ability of organisms to tolerate heat and cold is expected to be closely related to their maximum and minimum environmental temperatures. However, several studies have shown that whereas cold tolerance often correlates well with climatic variation along latitudinal or elevational gradients, heat tolerance remains relatively invariant (Bujan et al., 2020; Diamond & Chick, 2018). This difference in variability between maximum and minimum critical thermal limits has led to the niche asymmetry hypothesis (NAH), which proposes that, unlike cold tolerance, heat tolerance is largely conserved across lineages rather than varying with experienced temperature (Araújo et al., 2013; Hoffmann et al., 2013; but see Herrando‐Pérez et al., 2020).

Phylogenetic conservatism in upper thermal tolerances has important consequences for species living in places where temperatures are close to their upper thermal limits, since these species have reduced evolutionary potential for adaptation to global warming (Araújo et al., 2013; Bennett et al., 2021; Hoffmann et al., 2013). Warming vulnerability appears to be greatest for tropical ectotherms (Deutsch et al., 2008; Diamond, Sorger, et al., 2012), because latitudinal variation in their maximum critical thermal limits is much smaller than the latitudinal variation in environmental temperatures (Deutsch et al., 2008). However, thermal variation across latitude can be swamped by thermal variation at local scales due to variation in exposure to direct insolation (Scheffers et al., 2013). For example, temperature in the canopy of tropical forests can be markedly higher than on the ground (Leahy et al., 2021; Scheffers et al., 2013). Similarly, temperature on the ground in open habitats can be substantially higher than in forest. Species living in the same macroclimatic region can therefore differ widely in the microclimatic conditions that they are exposed to (Baudier et al., 2015; Herrando‐Pérez et al., 2020; Kirchner et al., 2025). This means that species assemblages living in close proximity may differ in their degree of vulnerability to global warming, and this may not necessarily reflect the difference in their upper critical thermal limits (Leahy et al., 2022).

Here, we compare the warming tolerance of ants in savanna and adjacent semideciduous forest and between different vertical strata in Brazil's Cerrado biome, where maximum temperatures have increased by 4°C over recent decades and are predicted to increase another 2°C by 2050 (Hofmann et al., 2021). Although ants have been often used as model systems in studies of thermal tolerance (Nascimento et al., 2022; Parr & Bishop, 2022; Roeder et al., 2021), critical thermal limits are known for only a tiny fraction (<3%) of species, most of which live in the temperate zone (Nascimento et al., 2022). Information about the warming vulnerability (as defined above) of this ecologically dominant insect group is even more limited and is not available for any Cerrado ant species. The same is true for other groups of Cerrado ectotherms, as existing studies have considered only potential future changes in species distributions (Alves‐Ferreira et al., 2022) or habitat‐related differences in thermal tolerances (Silva et al., 2020), not warming vulnerability.

Our study began by determining the extent to which differences in the upper and lower thermal limits of ant assemblages associated with different habitats and vertical strata conformed to the TAH or NAH hypotheses. According to the TAH, one would expect that the difference in the maximum, minimum, and in the amplitude (range) of the thermal limits of ant assemblages from different habitats or strata would closely reflect the differences in maximum, minimum, and range of temperatures that are experienced. However, according to the NAH, differences in the upper thermal limits would be expected to be much smaller than microclimatic differences. We then evaluated the degree of phylogenetic signal in the critical thermal limits of the focal ant assemblages, testing if eventual differences between habitats and vertical strata in these limits were maintained after using phylogenetically corrected analyses. Much of the ant fauna of Brazilian savannas has a forest origin, so that the two faunas share many lineages (Andersen & Vasconcelos, 2022; Vasconcelos et al., 2018). In contrast, in both savanna and forest, there are large differences in ant faunal composition at the genus level between the arboreal and the ground strata (Vasconcelos et al., 2023). Finally, using microclimatic data obtained in the study area, we determined the extent to which ant assemblages from different habitats and strata differ in their degree of vulnerability to global warming. We predicted that differences in experienced temperatures among habitats and vertical strata, more so than variation in critical thermal limits, drove differences among ant assemblages in warming vulnerability.

## METHODS

### Study area and ant sampling

This study was conducted at the Reserva Ecológica do Panga (REP), a 404‐ha reserve located 30 km south of Uberlândia, Minas Gerais, Brazil (19° 10′ S, 48° 23′ W). The mean annual temperature of the region is 22°C, and the mean annual rainfall is 1650 mm. The REP is located within the Cerrado biome, which is characterized by a mosaic of vegetation types, including savannas, grasslands, and forests (Cardoso et al., 2009).

We collected ants for thermal tolerance tests in 2022 (February) and again in 2025 (January–March). Ants were collected in two habitats of the reserve: savanna and semideciduous forest (Appendix S1: Figure S1). The latter has a relatively closed tree canopy (12–18 m in height) whereas the savanna has a much sparser tree cover, formed by trees usually not taller than 6 m in height (Cardoso et al., 2009). Ants were hand collected in both habitats during the day and the night to maximize the number of species sampled since previous studies indicate that night foraging is a way to avoid extreme daytime temperatures, notably by ground‐nesting ants from more open habitats (Cerdá et al., 1998; Marsh, 1988). Whenever necessary we placed sardine baits on the ground or in trees to generate ant activity and collected the ant workers that were approaching the bait but that were not on it. In 2025, we also collected ants from ca. 20 samples of 1 m^2^ of leaf‐litter from each habitat using a Winkler extractor. All ant specimens collected were kept in alcohol after the thermal tolerance tests. In the lab, a representative specimen from each sample was dry‐mounted for subsequent identification using available taxonomic keys or by comparing each specimen with specimens previously identified by ant taxonomists and deposited at the Zoological Collection of the Federal University of Uberlândia (UFU). Specimens for which a species‐level identification was not possible received a morphospecies code, which was the same used in UFU's collection (and in previous publications from our research group; Neves et al., 2024; Vasconcelos et al., 2018). In total, we collected ants from 155 colonies, belonging to 94 ant species from 34 genera and 8 subfamilies. Thirty‐eight species were found only in the savanna, 30 only in the adjacent semideciduous forest, and 26 species were found in both habitats (Appendix S1: Table S1). In the last case, separate thermal tolerance tests were performed with individuals collected in each habitat. Hereafter, we refer to as “savanna” and “forest” species all those found in savanna and forest respectively, whether exclusively or not.

Collected species or morphospecies were classified as arboreal or ground‐dwelling based on (a) known nesting/foraging specialization (e.g., species of *Cephalotes*, *Pseudomyrmex* and *Azteca* as arboreal specialists), (b) information on the relative frequency that different ant species or morphospecies were collected in ground versus arboreal traps during previous ant surveys in the Cerrado (Neves et al., 2024; Vasconcelos et al., 2018), and (c) natural history data available at the AntWeb platform (www.antweb.org). Of the 94 total species or morphospecies, 68 were classified as ground‐dwelling and 26 as arboreal.

### Thermal tolerance

The CT_max_ and CT_min_ of sampled individuals were determined within 5‐h of collection using a Kasvi model K80‐S01/02 Dry Bath and a Loccus model DB‐HC Dry Bath, respectively. In each test, 10 ant workers (for polymorphic or dimorphic species only minor workers were tested) of each species were placed individually in a 2‐ml microcentrifuge vial sealed with a small cotton ball and placed randomly in the dry bath equipment. The initial temperature of the CT_max_ test was 36°C, which was increased by two degrees every 10 min until death or loss of muscle coordination of the workers. For CT_min_, the initial temperature was 16°C, which was decreased by two degrees every 10 min until death or loss of muscle coordination of the workers. We considered the CT_max_ and CT_min_ of the species as the mean temperature of death or permanent loss of muscle coordination of all the tested workers. CT_range_ was calculated as the difference between CT_max_ and CT_min_.

### Phylogenetic signal

Analysis of phylogenetic signal was based on a species‐level phylogeny built with DNA sequences of ultraconserved elements of 357 Cerrado ant species (Neves et al., 2024). Prior to conducting the analysis, the phylogenetic tree was pruned to include only the ant species or morphospecies collected in the present study (Appendix S1: Figure S2). We calculated mean CT_max_, CT_min_, and CT_range_ for each species and used these data to evaluate the degree of phylogenetic signal for these traits using Blomberg's K statistic (Blomberg et al., 2003). The statistical significance of observed *K*‐values was assessed through randomization tests (*n* = 999 permutations). A *K*‐value greater than one implies that close relatives are more similar to each other than expected under Brownian motion (Blomberg et al., 2003). A *K*‐value close to 0 is indicative of no phylogenetic signal, whereas significant values of *K* ranging from 0.4 to 0.7 are often regarded as indicative of a moderate signal (Blaimer et al., 2015). We used the R package “phytools” (Revell, 2024) to compute phylogenetic signals and statistical significance.

### Microclimate

We used Hobbo pendant MX2202 dataloggers to determine daily maximum and minimum ambient air temperatures in the savanna and forest habitats, both on ground (ca. 0.5 m above ground) and in the canopy (ca. 6 m above ground in the savanna, and 10–12 m in the forest, which are the mean height of the canopy in each environment; Cardoso et al., 2009). Ambient air temperatures were recorded at 10‐min intervals from 21 December 2024 to 20 March 2025 (i.e., austral summer). We set one datalogger in each habitat/stratum combination (*n* = 4) moving its location to a new randomly selected point (~1 km apart) within the same habitat/stratum at about every 30 days. To avoid overheating, the dataloggers were put inside radiation shields (Holden et al., 2013; Terando et al., 2018).

### Warming tolerance

Warming tolerance was used as a measure of species' vulnerability to global warming, since it provides an estimate of how much the environment can warm before an organism reaches physiological failure or death (Diamond, Sorger, et al., 2012). Warming tolerance was calculated as “CT_max_ *—ambient temperature*,” where ambient temperature is the mean daily maximum ambient air temperatures of the habitat /stratum it was associated with (as recorded during the austral summer of 2024/5).

### Statistical analysis

Statistical analyses were conducted in R (R Core Team, 2024). We assessed differences in CT_max_ and CT_min_ between habitats, vertical strata, and sampling year (2022 and 2025) using three‐way ANOVAs. To account for phylogenetic nonindependence, we also performed phylogenetic ANOVAs using the function “phylANOVA” in the R package “phytools” (Revell, 2024). For species we were able to record CT_max_ and/or CT_min_ in both sampling years, a mean value was calculated. We compared the critical thermal tolerances between arboreal and ground‐dwelling species as well as between species exclusively found in the savanna or in the forest. It was not possible to include in this latter analysis the species that were found in both habitats since the same species cannot appear twice in the phylogeny. For these species, habitat‐related differences in thermal tolerances were assessed using a paired *t*‐test.

Differences in microclimate between habitats and vertical strata (over the summer of 2024/5) were assessed using a randomized‐block ANOVA, in which sampling day was treated as a blocking (random) factor. The response variable represented the maximum or the minimum temperatures recorded on each sampling day (*n* = 90). Finally, differences in warming tolerance between ant assemblages in different habitats and vertical strata were evaluated using a two‐way ANOVA of means of component species. There were no differences in CT_max_ between 2022 and 2025 (*F*_1,142_ = 0.02, *p* = 0.89), and so data were combined for analyses of warming tolerance. Whenever a significant interaction between main factors was detected, a multiple comparison test was performed using the Tukey method.

## RESULTS

CT_max_ was recorded for a total of 92 species, CT_min_ for 93, and both CT_max_ and CT min for 91 species, considering both the forest and savanna habitats and the arboreal and ground strata (Appendix S1: Table S1). Among these species, CT_max_ varied from 37.8°C in *Hypoponera* sp. 4 to 47.4°C in *Cephalotes depressus*, and CT_min_ varied from 3.4°C in *Camponotus bonariensis* to 14°C in *Hypoponera* sp. 3, whereas the amplitude of thermal tolerances (CT_range_) varied from 24°C in *Hypoponera* sp. 3 to 42.9°C in *Camponotus bonariensis*.

### Phylogenetic signal

There was a significant phylogenetic signal for CT_max_, CT_min_, and CT_range_, and all observed *K*‐values (CT_max_: *K* = 0.63, *p* < 0.001; CT_min_: *K* = 0.50, *p* < 0.001; CT_range_: *K* = 0.73, *p* < 0.001) can be regarded as indicative of a moderate phylogenetic signal (cf. Blaimer et al., 2015). Both CT_max_ and CT_range_ were lower for species of the subfamily Ponerinae than for species of Dolichoderinae, Formicinae, Myrmicinae, and Pseudomyrmecinae, whereas CT_min_ was higher for species of Ponerinae than for Dolichoderinae, Myrmicinae, and Formicinae (Appendix S1: Figure S3).

### Influence of habitat type and vertical stratum

**FIGURE 1:**
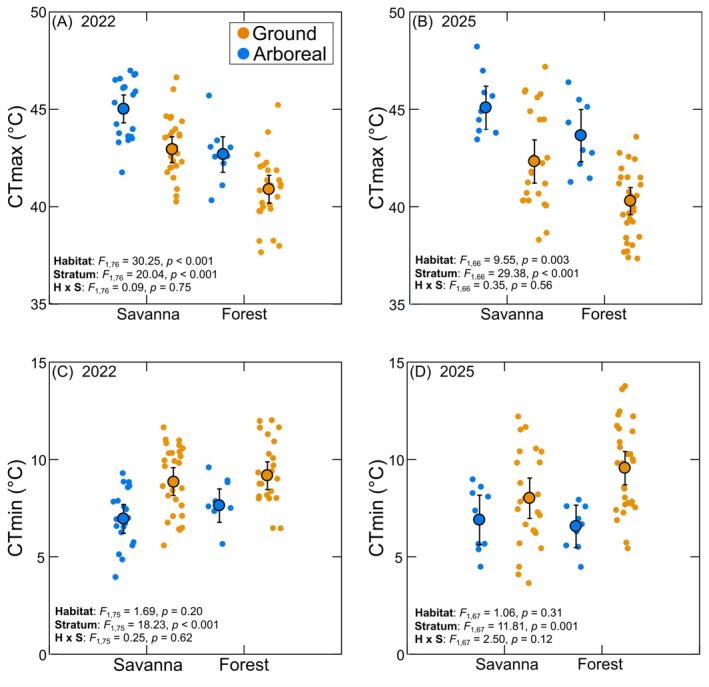
Influence of habitat type and vertical stratum on the critical thermal maxima (CT_max_) and minima (CT_min_) of Neotropical ant assemblages as measured in the austral summers of 2022 and 2025. Large circles represent mean values (±95% CI), while small circles represent data from individual ant species.

The mean CT_max_ was higher for savanna than for forest ants (*F*_1,142_ = 33.59, *p* < 0.001) and higher for arboreal than for ground species (*F*_1,142_ = 55.85, *p* < 0.001), with no interaction between habitat type and vertical stratum (*F*_1,142_ = 0.10, *p* = 0.75). Similar results were obtained in the two study periods (i.e., there was no interaction between sampling year and habitat; *F*_1,142_ = 0. 35, *p* = 0.56, or between sampling year and vertical stratum; *F*_1,142_ = 2.78, *p* = 0.097). In 2022, mean CT_max_ was 2.49 ± 0.83°C (mean ± 95% CI) higher among the species collected in the savanna habitat than in those from the adjacent forest, and this figure was 2.02 ± 1.19°C in 2025 (mean values including data from all vertical strata) (Figure 1A,B). Mean CT_max_ was higher among the arboreal than among the ground species in both habitat types in the two study periods (2022: 2.42 ± 0.83°C higher on average; 2025: 3.18°C ± 1.06 higher; mean values including data from the two sampled habitats) (Figure 1A,B).

CT_min_ did not differ between habitats (*F*_1,142_ = 2.62, *p* = 0.11) but was higher for arboreal than for ground species (*F*_1,142_ = 28.61, *p* < 0.001). There was no interaction between the effects of habitat and stratum on CT_min_ (*F*_1,142_ = 1.13, *p* = 0.29), and neither of these factors presented a significant interaction with sampling year (year × habitat: *F*_1,142_ = 0. 02, *p* = 0.88, year × stratum: *F*_1,142_ = 1. 13, *p* = 0.29) (Figure 1C,D). Regardless of the habitat type, arboreal species were, on average, more cold‐tolerant than the ground species; in 2022, the mean CT_min_ was 1.81 ± 0.72°C lower, whereas in 2025, it was 2.12 ± 0.75°C lower in the arboreal than in the ground species (Figure 1C,D).

CT_range_ was significantly greater for the savanna than for the forest fauna (savanna: 35.66 ± 0.85°C, forest: 32.41 ± 0.95°C; *F*_1,137_ = 17.66, *p* < 0.001; means including both arboreal and forest species) and greater for the arboreal than for the ground fauna (arboreal 37.30 ± 0.70°C, ground: 32.61 ± 0.80°C; *F*_1,137_ = 52.54, *p* < 0.001; means including species from savanna and forest). There was no interaction between the effects of habitat type and stratum on CT_range_ (*F*_1,137_ = 0.29, *p* = 0.59), with similar findings observed at the two study years (sampling year × habitat; *F*_1,137_ = 0.04, *p* = 0.85; sampling year × vertical stratum, *F*_1,142_ = 1.54, *p* = 0.22). CT_range_ was significantly correlated with both CT_max_ (*r* = 0.92, *p* < 0.001) and CT_min_ (*r* = −0.89, *p* < 0.001), but more strongly so with the former than with the latter. As analysis with CT_range_ presented results qualitatively very similar to those with the other critical thermal tolerance measures, hereafter we report solely results involving either CT_max_ or CT_min_.

**FIGURE 2:**
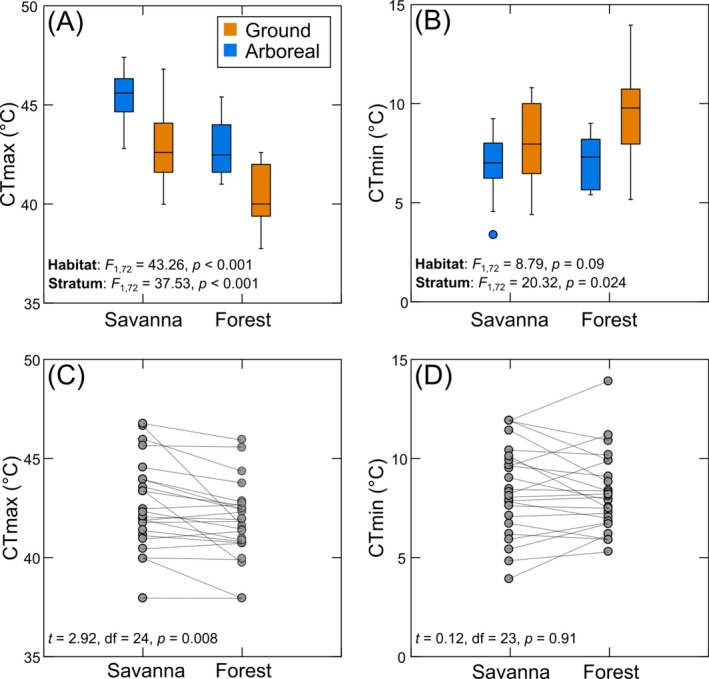
(A, B) Influence of habitat type and vertical stratum on the critical thermal maxima (CT_max_) and minima (CT_min_) among ant species that were exclusively found in the savanna or in forest (data from the two study years combined). Statistical values account for the phylogenetic nonindependence of the data (phylANOVAs comparing habitats and strata separately). (C, D) CT_max_ and CT_min_ for species found in both the savanna and forest habitats. Lines connect data from the same species in different habitats.

Accounting for phylogenetic nonindependence did not substantially change these results. Combining data from the two study years, we found that mean CT_max_ of species exclusively found in the savanna was 3.11 ± 0.93°C higher than that of species found only in the forest (Figure 2A) and that mean CT_max_ of arboreal species was 2.91 ± 0.94°C higher than that of ground species (Figure 2A). Mean CT_min_ did not differ significantly between species exclusively found in different habitats (Figure 2B), but it was 1.96 ± 0.87°C lower among arboreal than ground species (Figure 2B).

For species found in both savanna and forest, mean CT_max_ was 0.82 ± 0.58°C higher for those in savanna than in forest (Figure 2C), whereas there was no significant difference in mean CT_min_ among habitats (Figure 2D).

### Microclimate

**FIGURE 3:**
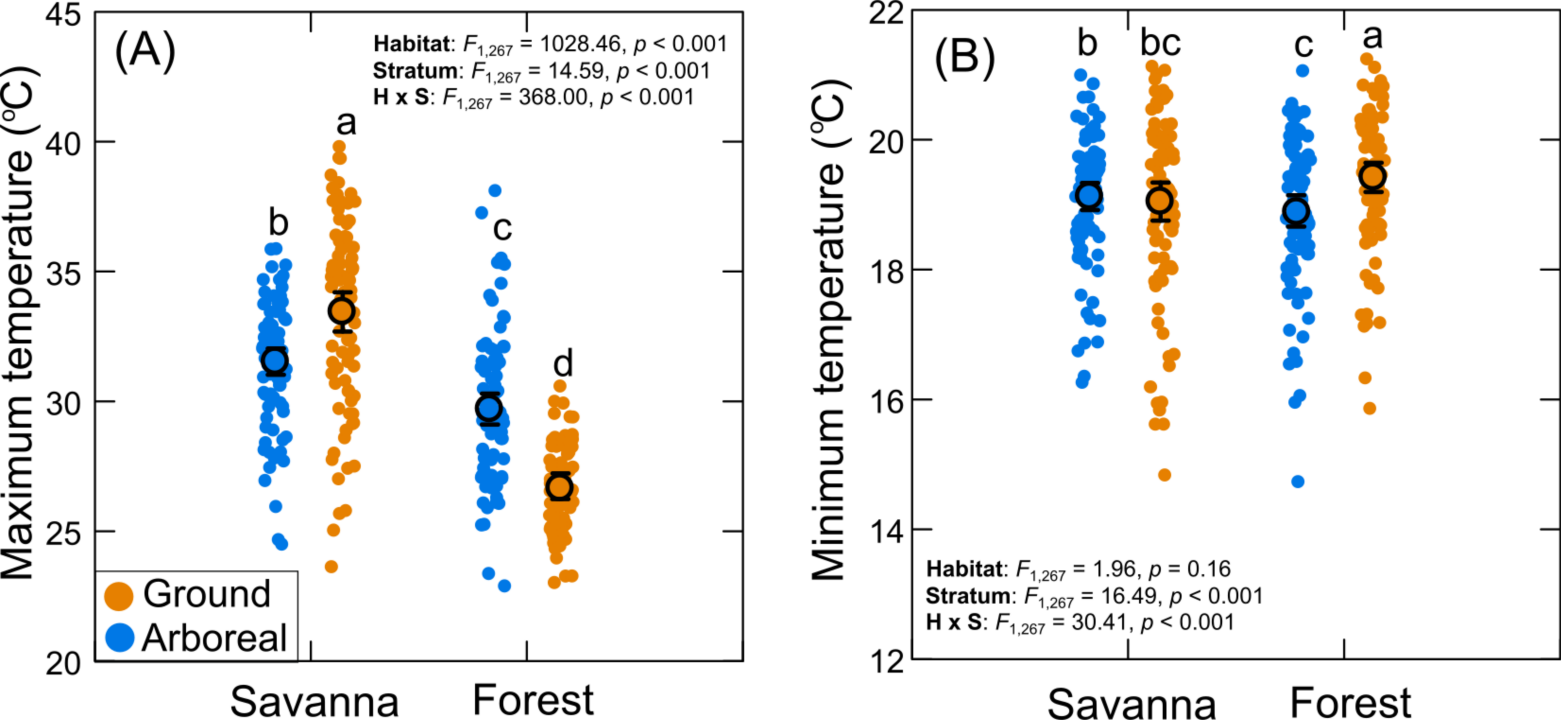
Differences in maximum (A) and minimum (B) daily temperatures (over the austral summer of 2024/5) between the arboreal and ground strata of the savanna and semideciduous forest habitats. Different letters above the data points represent differences in mean values among different habitat/stratum categories. For both panels, see Appendix S1: Table S2 for full results of post hoc pairwise comparisons.

The mean maximum ambient temperature was significantly higher in savanna than in forest (savanna: 32.4 ± 0.47°C, forest: 28.2 ± 0.41°C). However, there was also a significant interaction between habitat type and vertical stratum on mean maximum ambient temperature (Figure 3). In the forest, the mean maximum temperature was 3.05 ± 0.68°C higher in the canopy than on the ground, but in the savanna, mean maximum temperature was 2.04 ± 0.90°C higher on the ground. Furthermore, the magnitude of the difference in mean maximum temperature when comparing savanna and forest was higher in the ground (6.79 ± 0.82°C) than in the arboreal stratum (1.71 ± 0.78°C) (Figure 3A).

There was no difference in mean daily minimum temperatures between savanna and forest (Figure 3B). However, an interaction between habitat and stratum was detected. Within the forest, minimum daily temperatures were on average slightly (0.52 ± 0.33°C) lower in the canopy than on the ground, whereas in the savanna, the difference between vertical strata in mean minimum temperatures was negligible (0.08 ± 0.36°C) (Figure 3B).

Analysis of the variation in the amplitude (range) of temperatures across habitats and strata produced results that largely mirrored those observed with maximum temperatures. There was an effect of habitat (*F*_1,267_ = 886,3, *p* < 0.001), strata (*F*_1,267_ = 24.9, *p* < 0.001), as well as an interaction between these two factors (*F*_1,267_ = 381.8, *p* < 0.001). In the savanna, temperature range was 1.17 times greater on ground than in trees (arboreal: 12.34 ± 0.62°C; ground: 14.45 ± 0.94°C), whereas in the forest, temperature range was 1.49 times greater in the arboreal than in the ground stratum (arboreal: 10.85 ± 0.72°C; ground: 7.29 ± 0.47°C).

### Warming tolerance

**FIGURE 4:**
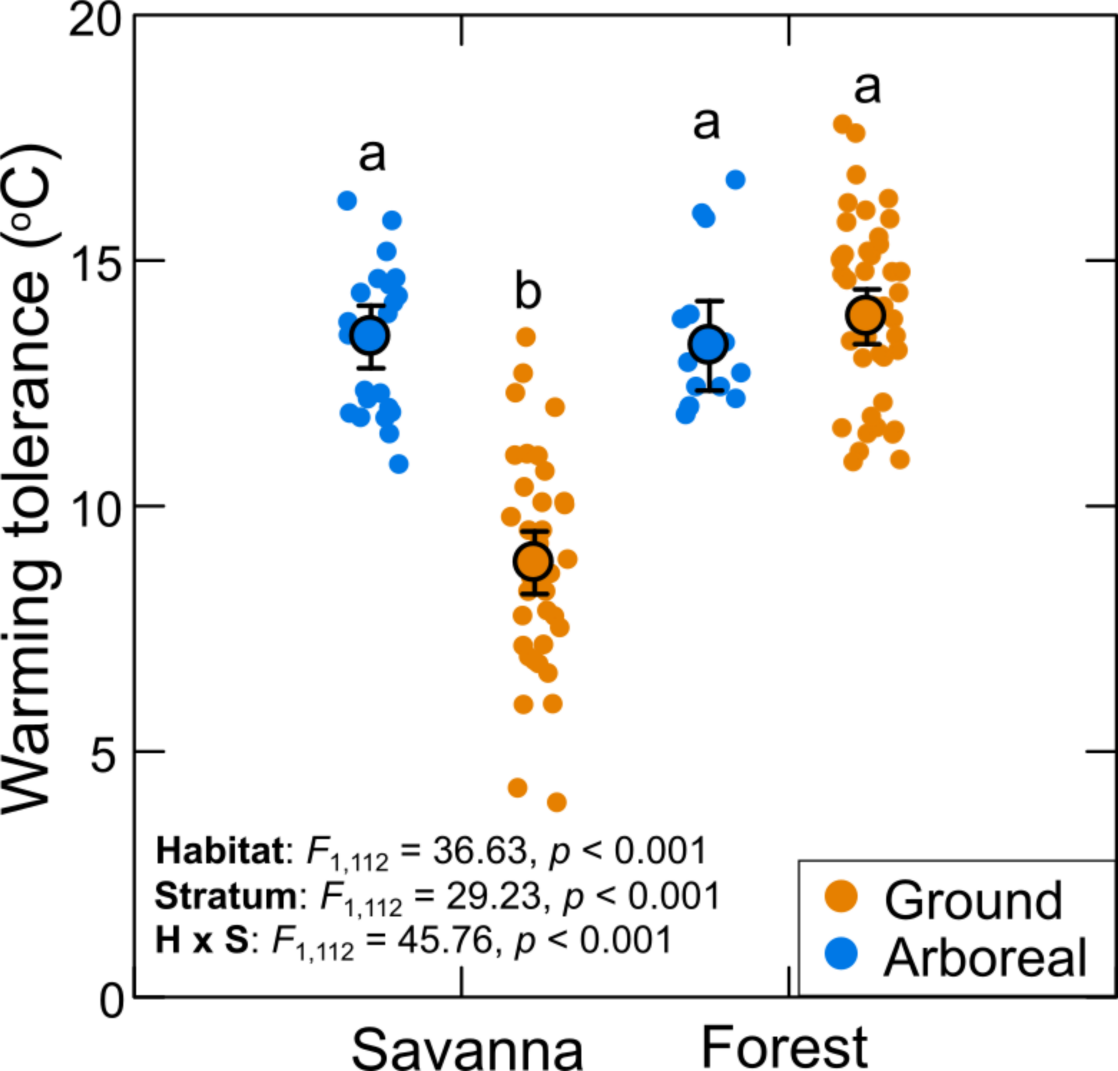
Warming tolerances of ant assemblages from different habitats and vertical strata. Warming tolerance represents the difference between a species' CT_max_ and the average maximum air temperature of its foraging/nesting habitat and stratum. Large circles represent mean values (±95% CI), while small circles represent data from individual ant species. Different letters above the data points represent differences in mean values among different habitat/stratum categories. See Appendix S1: Table S3 for full results of post hoc pairwise comparisons.

Warming tolerance (i.e., CT_max_ minus mean maximum ambient temperature) ranged from 4.45 to 17.74°C overall. Considering all species, on average, warming tolerance was 3.25 ± 0.89°C higher among savanna than among forest ants and 2.06 ± 1.13°C higher among arboreal than among ground ants. However, there was a highly significant interaction between habitat and stratum on warming tolerance (Figure 4). Mean warming tolerance was about 14°C for assemblages of both strata in the forest and for the arboreal assemblage in savanna, but it was considerably lower (8.92°C) for the ground assemblage in savanna. Warming tolerance was <10°C for 29 of the 41 ground species in savanna, but it was >10°C for all arboreal species in savanna and for all forest species (Figure 4).

If we take into account the highest rather than mean maximum temperature recorded in each habitat and stratum over the summer of 2024/5 (arboreal forest = 37.7°C, arboreal savanna = 36.2°C, ground forest = 30.37, ground savanna = 39.8°C) as the measure of ambient temperature, then warming tolerances ranged from −1.83 to 14.03°C overall. In total, 17 species presented a warming tolerance <2°C and all were ground savanna species.

## DISCUSSION

Warming tolerance is a product of both critical thermal maximum and exposure temperature, and the latter can show marked local variation due to variation in direct insolation. Our study addresses variation in ant warming tolerance between adjacent savanna and forest, and between ground and arboreal strata, in the Brazilian Cerrado biome, which has already experienced substantial rises in mean temperature over recent decades (Hofmann et al., 2021). This is the first study that addresses variation among intact tropical ant assemblages in relative warming vulnerability by integrating critical thermal limits and microclimate, considering both habitat type and vertical stratification.

### Critical thermal limits

We first evaluated the extent to which horizontal (across habitats) and vertical (between strata) variation in the critical thermal limits of Neotropical ants conforms to the TAH (Kaspari et al., 2015) and the NAH (Araújo et al., 2013; Herrando‐Pérez et al., 2020) hypotheses. Previous studies, with a variety of ectotherm taxa, suggest that both hypotheses are valid, depending on the scale of observations. While the NAH usually applies to geographic scales (Araújo et al., 2013; Leahy et al., 2022; Pintanel et al., 2025), that is not often the case for the TAH whose support often derives from local scale studies such as those comparing different microhabitats (Brusch IV et al., 2016; Duarte et al., 2012; Kaspari et al., 2015; Leahy et al., 2022; Pintanel et al., 2022; Silva et al., 2020).

Our study shows that savanna ants in both the arboreal and ground strata experience higher and more variable temperatures and present both higher CT_max_ and CT_range_ than those living in the forest. In addition, we found that the magnitude of the difference in maximum air temperatures between the forest canopy and the forest floor (3.05°C) was similar to that in CT_max_ between arboreal and ground forest species (2.61°C). Finally, the difference in mean maximum temperatures between the savanna and the forest canopies (1.71°C) closely matched the difference in CT_max_ between arboreal savanna and arboreal forest ants (1.98°C). Overall, these findings are consistent with the TAH rather than the NAH, and they reinforce the notion that microhabitats can shape the heat tolerance of ectotherms (Kaspari et al., 2015; Leahy et al., 2022; Pintanel et al., 2025).

However, some of the observed relationships between mean maximum temperature and mean CT_max_ were not consistent with the TAH. Despite experiencing higher (~2°C on average) and more variable temperatures, ground savanna ants had lower CT_max_ and lower CT_range_ than those living in savanna trees. Further, the difference in mean maximum air temperatures on the ground (6.79°C) between savanna and forest was 3.6 times greater than the difference in CT_max_ between ground savanna and ground forest ants, a finding that is more consistent with the NAH than with the TAH. Finally, we found that although arboreal ants in both forest and savanna had lower mean CT_min_ than did the ground ants, the differences in average minimum temperatures between these two strata were negligible. This is not consistent with either the TAH or NAH.

### Phylogenetic signal

Previous studies on ectotherms indicate that CT_min_ is a more labile trait than is CT_max_ (Diamond & Chick, 2018; Hoffmann et al., 2013; Nascimento et al., 2022; von May et al., 2019). However, here, we detected a moderate level of phylogenetic conservatism in both the lower and upper thermal tolerances of Cerrado ants (albeit Blomberg's *K* was not as high in the former than in the latter; *K* = 0.50 cf. 0.63). Accordingly, species from more basal clades (which are predominantly or exclusively terrestrial), notably Ponerinae, were less heat‐ and cold‐tolerant than those from predominantly arboreal clades, such as Pseudomymecinae, *Camponotus* (Formicinae) and *Azteca* (Dolichoderinae). The fact that many arboreal ant clades showed high thermal tolerances (both lower and upper tolerances) may well be associated with the fact that nests of arboreal ants, notably those established in small tree branches, are less buffered against thermal fluctuations (temperature extremes) than those of ground‐nesting ants (Kirchner et al., 2025). This interpretation is in line with a study on solitary bees showing that the effects of climate change on larval development are more pronounced for cavity‐nesting than for ground‐nesting species (Herrera et al., 2023).

### Warming tolerance

Our final aim was to compare the warming tolerance (a proxy of vulnerability to global warming) of arboreal and ground‐dwelling ants from adjacent savanna and forest. Previous studies have indicated that ants living in the canopy of lowland tropical rainforests are those at most risk from global warming (Câmara et al., 2024; Diamond, Sorger, et al., 2012; Leahy et al., 2022). This is because the difference in ambient temperatures between the canopy and the ground of tropical rainforests is much larger than the difference in CT_max_ between species living in different vertical strata (Leahy et al., 2021; Leahy et al., 2022). In our study, by contrast, forest ants associated with different vertical strata showed similar levels of warming tolerance. We attribute these contrasting results to differences in the steepness of vertical climatic gradients of temperature. Canopy trees in tropical rainforests are much taller than those in semideciduous forest (Oliveira‐Filho & Ratter, 2002), such as ours, and thus, variation in air temperatures from the ground to the canopy is likely to be greater in the former (e.g., Leahy et al., 2021).

Furthermore, and in sharp contrast with the situation seen in the forest, ground ants in savanna were exposed to higher temperatures than those in trees, despite their lower heat tolerance. In addition, the observed increase in the CT_max_ of ground ants as one moves from the forest to the savanna was not high enough to overcome the strong difference in ground maximum temperatures between these two habitats. Together, these results strongly suggest that, in the Neotropics, savanna ground ants are more vulnerable to global warming than are ants on the forest floor or arboreal ants more generally. We suggest that the higher susceptibility of the Cerrado savanna ground ant fauna to global warming is linked to its evolutionary history in association with the Amazon and Atlantic Forest faunas. The ground ant fauna of the Cerrado savanna contains few highly thermophilic species, which is in sharp contrast with the Australian savanna ant fauna that has evolved in association with a desert biome (Andersen & Vasconcelos, 2022).

Over the summer of 2024/5, we recorded ambient air temperatures that are already higher than the CT_max_ of two ground savanna species (*Anochetus inermis* and *Apterostigma* sp. 3) in 13% of the sampling days (for a period of 10–110 min in each day). On the other hand, when warming tolerance was calculated based on the maximum absolute temperature recorded over our study period (rather than on the mean of maximum values), we found that nearly half (42%) of the savanna ground species have a warming tolerance <2°C, and therefore below the forecasted increase of 2°C in Cerrado's maximum temperatures by 2050 (Hofmann et al., 2021). Furthermore, under direct insolation, temperatures of foraging surfaces can be far higher than the ambient air temperatures we recorded. In fact, surface ground temperatures of 60°C or more have been recorded both in the Cerrado and in the adjacent Caatinga (Camacho et al., 2015; Vitt et al., 2007). These values surpass the CT_max_ of all ground ants we tested. Such greater warming vulnerability of ground savanna ants can help to explain why the reversed latitudinal gradient of ant species richness in the Cerrado is much steeper for the ground than for the arboreal assemblages (Vasconcelos et al., 2023), such that ant diversity on the ground decreases faster than in trees as one moves from the colder, higher latitude regions of the Cerrado to the hotter, lower latitude regions.

Although ant endemism in the Cerrado is relatively low, many of the known endemic species (e.g., *Ectatomma planidens*, *Mycetagroicus cerradensis*, *Sericomyrmex maravalhas*) are ground species associated with open savanna (Brandão & Mayhé‐Nunes, 2001; Ješovnik & Schultz, 2017). Conservation of such species may therefore be challenging, and not only because rates of habitat conversion to agriculture in the Cerrado are rampant (Strassburg et al., 2017). It is also because a misguided fire suppression policy has transformed many open savannas into dense savannas or dry forests (Abreu et al., 2017; Durigan & Ratter, 2016).

Opportunities to establish new areas for conservation of Cerrado's biodiversity are highest where the natural vegetation has not yet been converted into human uses, which is mostly in the north of the Cerrado (Vieira et al., 2018). However, this is where species richness is lowest for ants (Vasconcelos et al., 2018; Vasconcelos et al., 2023), and where the impacts of climate change on ground ants may be greatest given that this is already the hottest region of the biome. As revealed by a recent study involving a wide range of taxa, the rapid evolution of upper thermal tolerances in ectotherms is highly unlikely (Bennett et al., 2021), meaning that the possibilities of physiological adaptation by these organisms to a fast‐changing climate are also small. Therefore, Neotropical savanna ground ants will need to rely on behavioral adaptations to cope with climate change, such as increased foraging in shaded microhabitats or during nighttime.

## CONCLUSION

Overall, our results show that vulnerability to global warming cannot be adequately predicted based on heat tolerance alone (Leahy et al., 2022; Walters et al., 2018). Our findings add to a growing number of studies indicating that estimates of warming vulnerability that are based on macroclimatic information alone may produce results that are inconsistent with those based on microclimatic data (Anderson et al., 2022). We show that species can have very different vulnerability to global warming despite having a similar CT_max_ because they live in different habitats or vertical strata and therefore exposure to direct insolation. Integrating information on species' habitat preferences with microclimatic information of their preferred habitats, although often logistically complex, may be the only way to achieve a robust understanding of how these species will respond to a warmer world.

## CONFLICT OF INTEREST STATEMENT

The authors declare no conflicts of interest.

## Supporting information


Appendix S1.

## Data Availability

Data (Zuanon & Vasconcelos, 2025) are available in Zenodo at https://doi.org/10.5281/zenodo.17246327.
