# Supplementary material for: Neotropical ants are at greater risk from global warming in savanna than in adjacent forest
Source: Ecology. 2026 May 13;107:e70413. doi: 10.1002/ecy.70413 (PMC13172437; doi:10.1002/ecy.70413)
Supplement: Supplementary file 1 — Appendix S1. [file ECY-107-e70413-s001.pdf]

Appendix S1

Neotropical ants are at greater risk from global warming in savanna than in adjacent forest

Lino A. Zuanon, Karen C. Neves, Alan N. Andersen, Heraldo L. Vasconcelos

Ecology

Supporting Information

Figure S1. General view of the savanna (left) and semideciduous forest (right) habitats. Photo credit: Lino A. Zuanon.

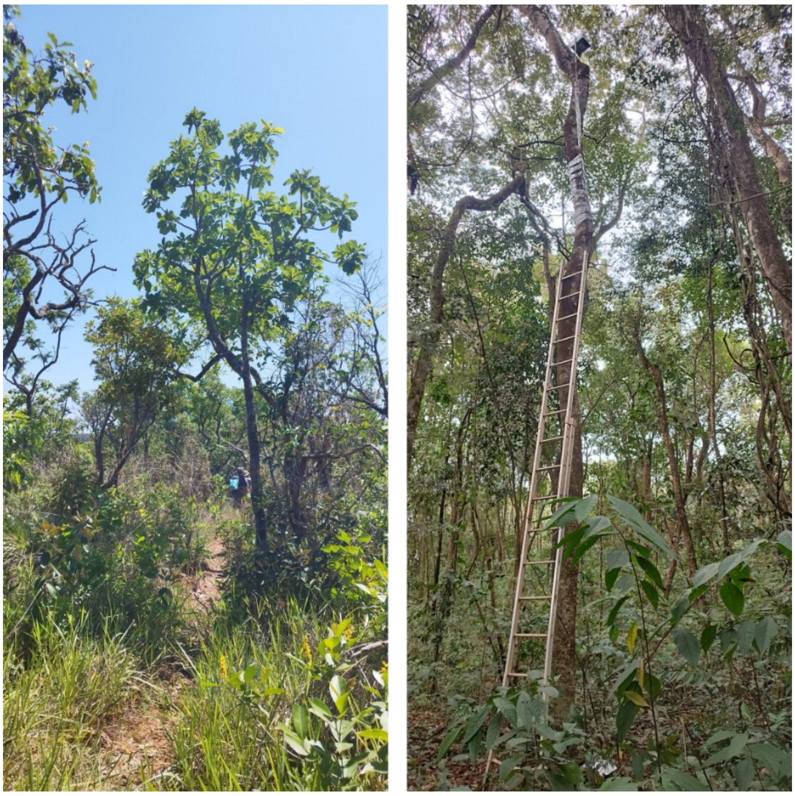

Figure S2. Phylogenetic tree of the 94 ant species analyzed in this study. The original topology, comprising 357 ant species, was pruned to include only the taxa relevant to the current analysis. Morphospecies codes represent those used in the ant collection form the Federal University of Uberlândia (UFU), Brazil.

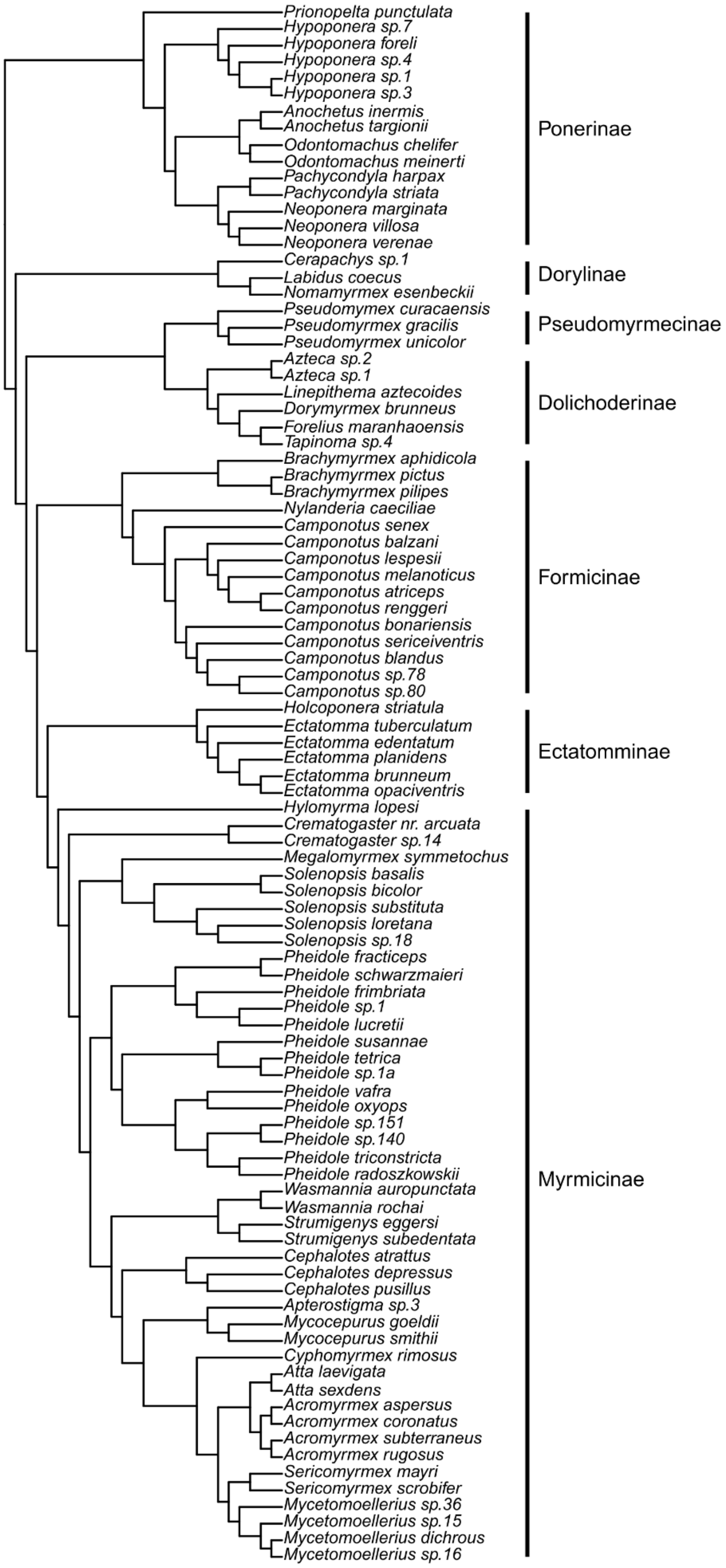

Figure S3. Critical thermal minima, maxima, and amplitude (range) of Neotropical ant species from different subfamilies. Different letters above the data points represent differences in mean values. Statistical analyses did not include the ant subfamily Amblyoponinae as it was represented by a single species.

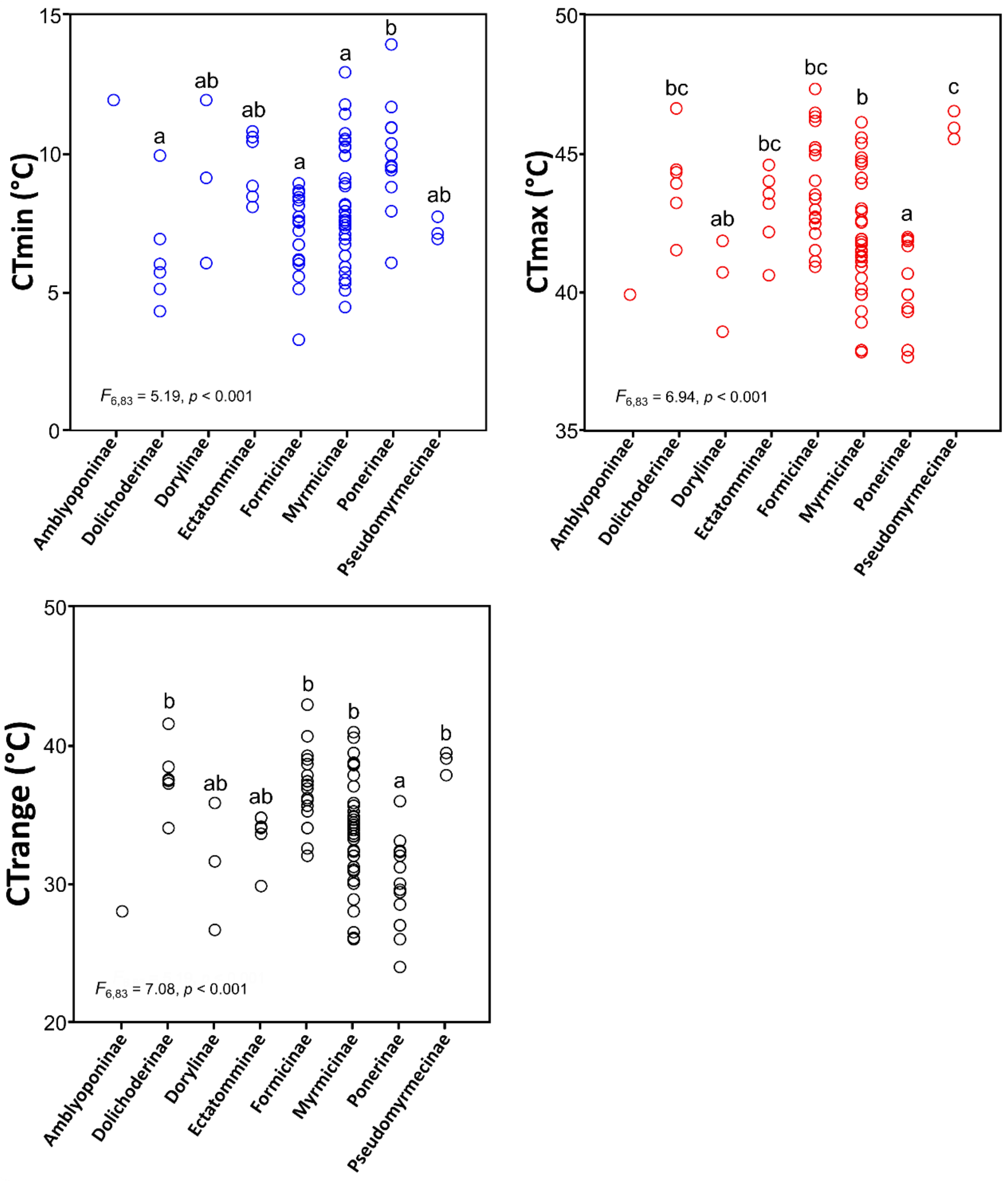

Table S1. List of the ant species for which measures of upper and/or lower critical thermal limits (CT_{max} and CT_{min}) were performed in 2022 and/or in 2025. Numbers represent the number of colonies tested. Morphospecies codes represent those used in the ant collection form the Federal University of Uberlândia (UFU), Brazil.

			2022		2025	
Habitat	Stratum	Ant species	CTmax	CTmin	CTmax	CTmin
forest	arboreal	*Azteca sp.2*	1	1	1	1
forest	arboreal	*Camponotus atriceps*	1	1	1	1
forest	arboreal	*Camponotus blandus*	0	0	1	1
forest	arboreal	*Camponotus lespesii*	1	1	0	0
forest	arboreal	*Camponotus melanoticus*	1	1	0	0
forest	arboreal	*Camponotus sp.78*	1	1	0	0
forest	arboreal	*Camponotus sp.80*	1	1	0	0
forest	arboreal	*Cephalotes pusillus*	0	0	1	1
forest	arboreal	*Ectatomma tuberculatum*	1	1	1	1
forest	arboreal	*Neoponera villosa*	0	0	1	1
forest	arboreal	*Solenopsis basalis*	1	1	1	1
forest	arboreal	*Solenopsis bicolor*	0	0	1	1
forest	arboreal	*Wasmannia rochai*	1	1	1	1
forest	ground	*Acromyrmex coronatus*	0	0	1	1
forest	ground	*Acromyrmex subterraneus*	1	0	1	1
forest	ground	*Anochetus inermis*	0	0	1	1
forest	ground	*Anochetus targionii*	1	1	1	1
forest	ground	*Apterostigma sp.3*	1	1	0	0
forest	ground	*Atta laevigata*	1	0	0	0
forest	ground	*Brachymyrmex aphidicola*	1	1	0	0
forest	ground	*Brachymyrmex pictus*	0	0	1	1
forest	ground	*Cerapachys sp.1*	0	0	1	1
forest	ground	*Crematogaster sp.14*	1	1	1	1
forest	ground	*Cyphomyrmex rimosus*	1	1	1	1
forest	ground	*Ectatomma edentatum*	1	1	1	1
forest	ground	*Holcoponera striatula*	1	1	1	1
forest	ground	*Hylomyrma lopesi*	0	0	1	1
forest	ground	*Hypoponera foreli*	0	0	1	1
forest	ground	*Hypoponera sp.1*	0	0	1	1
forest	ground	*Hypoponera sp.3*	0	0	1	1
forest	ground	*Hypoponera sp.4*	1	1	0	0
forest	ground	*Hypoponera sp.7*	0	0	1	1
forest	ground	*Megalomyrmex symmetochus*	0	0	1	1
forest	ground	*Mycetomoellerius sp.16*	1	1	0	0
forest	ground	*Mycocepurus smithii*	1	0	0	0
forest	ground	*Neoponera marginata*	1	1	1	1
forest	ground	*Neoponera verenae*	0	1	0	0
forest	ground	*Nomamyrmex esenbeckii*	1	1	0	0
forest	ground	*Nylanderia caeciliae*	1	1	0	0
forest	ground	*Odontomachus chelifer*	1	1	0	1
forest	ground	*Odontomachus meinerti*	1	1	1	1

			2022		2025	
Habitat	Stratum	Ant species	CTmax	CTmin	CTmax	CTmin
forest	ground	*Pachycondyla harpax*	1	1	1	1
forest	ground	*Pachycondyla striata*	1	1	0	0
forest	ground	*Pheidole frimbriata*	1	1	0	0
forest	ground	*Pheidole oxyops*	1	1	0	0
forest	ground	*Pheidole radoszkowskii*	1	0	0	0
forest	ground	*Pheidole sp.1*	0	0	1	1
forest	ground	*Pheidole sp.140*	1	1	0	0
forest	ground	*Pheidole susannae*	0	0	1	1
forest	ground	*Pheidole triconstricta*	1	1	1	1
forest	ground	*Prionopelta punctulata*	0	0	1	1
forest	ground	*Sericomyrmex mayri*	1	1	1	1
forest	ground	*Solenopsis sp.18*	1	1	0	0
forest	ground	*Strumigenys eggersi*	0	0	1	1
forest	ground	*Strumigenys subedentata*	0	0	1	1
forest	ground	*Wasmannia auropunctata*	0	0	1	1
savanna	arboreal	*Azteca sp.1*	1	1	1	1
savanna	arboreal	*Camponotus balzani*	1	1	0	0
savanna	arboreal	*Camponotus blandus*	1	1	1	1
savanna	arboreal	*Camponotus bonariensis*	1	1	0	0
savanna	arboreal	*Camponotus melanoticus*	1	1	0	0
savanna	arboreal	*Camponotus renggeri*	1	1	0	0
savanna	arboreal	*Camponotus senex*	1	1	1	1
savanna	arboreal	*Camponotus sericeiventris*	1	1	1	1
savanna	arboreal	*Cephalotes atrattus*	1	1	0	0
savanna	arboreal	*Cephalotes depressus*	1	1	0	0
savanna	arboreal	*Cephalotes pusillus*	1	1	1	1
savanna	arboreal	*Crematogaster nr. arcuata*	1	1	1	1
savanna	arboreal	*Ectatomma tuberculatum*	1	1	1	1
savanna	arboreal	*Neoponera villosa*	1	1	0	0
savanna	arboreal	*Pseudomymex curacaensis*	1	1	0	0
savanna	arboreal	*Pseudomyrmex gracilis*	1	1	0	0
savanna	arboreal	*Pseudomyrmex unicolor*	1	1	0	0
savanna	arboreal	*Solenopsis basalis*	1	1	1	1
savanna	arboreal	*Tapinoma sp.4*	1	1	0	0
savanna	arboreal	*Wasmannia rochai*	1	1	1	1
savanna	ground	*Acromyrmex aspersus*	1	1	0	0
savanna	ground	*Acromyrmex rugosus*	0	0	1	1
savanna	ground	*Acromyrmex subterraneus*	1	0	0	0
savanna	ground	*Anochetus inermis*	0	0	1	1
savanna	ground	*Apterostigma sp.3*	0	0	1	1
savanna	ground	*Atta laevigata*	1	1	1	1
savanna	ground	*Atta sexdens*	1	1	0	0
savanna	ground	*Brachymyrmex aphidicola*	1	1	0	0
savanna	ground	*Brachymyrmex pilipes*	0	0	1	1
savanna	ground	*Crematogaster sp.14*	0	0	1	1
savanna	ground	*Dorymyrmex brunneus*	1	1	1	1

			2022		2025	
Habitat	Stratum	Ant species	CTmax	CTmin	CTmax	CTmin
savanna	ground	*Ectatomma brunneum*	1	1	1	1
savanna	ground	*Ectatomma edentatum*	0	0	1	1
savanna	ground	*Ectatomma opaciventris*	1	1	0	0
savanna	ground	*Ectatomma planidens*	1	1	0	0
savanna	ground	*Forelius maranhaoensis*	1	1	0	0
savanna	ground	*Holcoponera striatula*	1	1	0	0
savanna	ground	*Labidus coecus*	1	1	0	0
savanna	ground	*Linepithema aztecoides*	0	0	1	1
savanna	ground	*Mycetomoellerius dichrous*	1	1	0	0
savanna	ground	*Mycetomoellerius sp.15*	0	0	1	1
savanna	ground	*Mycetomoellerius sp.36*	0	0	1	1
savanna	ground	*Mycocepurus goeldii*	1	1	1	1
savanna	ground	*Neoponera marginata*	1	1	0	0
savanna	ground	*Neoponera verenae*	1	1	0	0
savanna	ground	*Nomamyrmex esenbeckii*	0	1	0	0
savanna	ground	*Odontomachus meinerti*	0	0	1	1
savanna	ground	*Pachycondyla harpax*	1	1	0	0
savanna	ground	*Pheidole fracticeps*	0	0	1	1
savanna	ground	*Pheidole lucretii*	0	0	1	1
savanna	ground	*Pheidole oxyops*	1	1	0	0
savanna	ground	*Pheidole radoszkowskii*	1	1	1	1
savanna	ground	*Pheidole schwarzmaieri*	0	1	0	0
savanna	ground	*Pheidole sp.151*	0	0	1	1
savanna	ground	*Pheidole suzannae*	0	0	1	1
savanna	ground	*Pheidole tetrica*	0	1	0	0
savanna	ground	*Pheidole triconstricta*	1	1	1	1
savanna	ground	*Pheidole vafra*	1	1	0	0
savanna	ground	*Sericomyrmex scrobifer*	1	1	0	0
savanna	ground	*Solenopsis loretana*	0	0	1	1
savanna	ground	*Solenopsis sp.18*	1	1	0	0
savanna	ground	*Solenopsis substituta*	1	1	1	1
savanna	ground	*Strumigenys eggersi*	0	0	1	1
savanna	ground	*Wasmannia auropunctata*	1	1	0	0

Table S2. Full results from Tukey´s post-hoc pairwise comparisons of the differences in maximum and minimum daily temperatures (over the austral summer of 2024/25) between the arboreal and ground strata of the savanna and semideciduous forest habitats. SA, Savanna Arboreal. FA, Forest Arboreal. SG, Savanna Ground. FG, Forest Ground). In bold, differences that were significant.

	Maximum temperature					Minimum temperature				
Comparison	estimate	SE	t-ratio	df	p-value	estimate	SE	t-ratio	df	p-value
SA – FA	-1.71	0.187	9.112	267	< **0.0001**	0.2219	0.076	2.910	267	**0.0204**
SA – SG	-2.04	0.187	-10.864	267	< **0.0001**	0.078	0.076	1.029	267	0.7328
SA – FG	4.75	0.187	25.377	267	< **0.0001**	-0.294	0.076	-3.861	267	**0.0008**
FA – SG	-3.74	0.187	-19.976	267	< **0.0001**	-0.143	0.076	-1.881	267	0.2387
FA – FG	3.05	0.187	16.265	267	< **0.0001**	-0.516	0.076	-6.771	267	< **0.0001**
SA – FG	6.79	0.187	36.241	267	< **0.0001**	-0.373	0.076	-4.890	267	< **0.0001**

Table S3. Full results from Tukey´s post-hoc pairwise comparisons of the differences in warming tolerance of ant assemblages from different habitats (Savanna and Forest) and vertical strata (Arboreal and Ground). In bold, differences that were significant.

Comparison	estimate	SE	t-ratio	df	p-value
Savanna Arboreal – Forest Arboreal	0.272	0.651	0.418	112	0.9753
Savanna Arboreal – Savanna Ground	4.651	0.498	9.337	112	< **0.0001**
Savanna Arboreal – Forest Ground	-0.247	0.496	-0.497	112	0.9595
Forest Arboreal – Savanna Ground	4.379	0.581	7.533	112	< **0.0001**
Forest Arboreal – Forest Ground	-0.519	0.58	-0.895	112	0.8074
Savanna Ground – Forest Ground	-4.898	0.401	-12.215	112	< **0.0001**
